# Closed Incision Negative Pressure Therapy vs Standard of Care Dressing in Breast Surgery: A Systematic Review

**DOI:** 10.7759/cureus.24499

**Published:** 2022-04-26

**Authors:** Amos Nepacina Liew, Kylie Yen-Yi Lim, Jeremy Fuquan Khoo

**Affiliations:** 1 Department of Breast Surgery, Monash Health, Moorabbin, AUS; 2 Department of General Surgery, Monash Health, Dandenong, AUS; 3 School of Clinical Medicine, Princess Alexandra Hospital, Southside Clinical Unit, The University of Queensland, Woolloongabba, AUS

**Keywords:** closed incision negative pressure wound therapy, npt, cinpt, closed-incision npt, wound complication, surgical site infection, breast surgery, negative pressure therapy, negative-pressure wound therapy

## Abstract

The implementation of closed incision negative pressure therapy (CINPT) is widely seen in many surgical subspecialties including orthopaedics, vascular surgery, and abdominal surgery. However, research on its use in breast surgery is still in its infancy. We conducted a systematic review on the use of CINPT vs standard of care dressings (SOC) in wound management of post-operative breast surgery.

A literature search was conducted on PubMed, MedLine, and Google Scholar for studies that compared CINPT against SOC. Seven studies were included in this systematic review. The results of our systematic review have shown that CINPT has a positive outcome in reducing post-operative wound complication rates as compared to SOC dressings (commonly Steri-Strips and waterproof dressings), which was 1-8% vs 1-30% in CINPT and SOC, respectively. Furthermore, CINPT has the potential to confer additional cost-savings of up to USD218 per patient for a health institution with regards to reduced complications rates that might have required extended management.

The use of CINPT in breast surgery remains highly promising. It has many advantages over SOC, including better wound outcomes and added cost savings. Further studies are required to delineate the potential benefits in different sub-sets of patients.

## Introduction and background

Breast disease and breast cancer management form a major part of healthcare delivery, constituting one of the most frequent elective surgeries performed globally. Breast cancer itself is one of the most common cancers in Australia, with one in eight women at risk of developing breast cancer in their lifetime. An estimated 20,000 new cases were diagnosed in 2021 [1]. Around 38% of patients with breast cancer will end up having a mastectomy [2]. Despite the volume of breast surgery performed each year and the modernisation of sterilisation methods, equipment, and surgical technique, post-operative wound complications from breast surgery still occur in the range of 1 to 30% [3]. Post-operative wound complications contribute to higher patient and hospital-based costs, with a mean attributable cost of USD574 from surgical site infections (SSI) after breast surgery [4]. There are several contributing factors to wound complications post-operation, both modifiable and non-modifiable.

Closed incision negative pressure therapy (CINPT) has been marketed over the last 20 years and has revolutionised the care of post-operative wounds. The principles of CINPT involve the application of a porous interface over a closed incision, replacing a traditional surgical dressing, e.g., Steri-Strips™ (3M Company, Saint Paul, Minnesota, United States) and OPSITE (Smith & Nephew Medical Limited, Kingston upon Hull, United Kingdom). Negative pressure in a closed system is then applied over the pressure dressing with a disposable machine [5]. This theoretically holds incision edges together, removes exudate, and prevents external contamination, promoting increased collagen recruitment at the incision site and tensile strength as compared to the traditional standard of care dressings (SOC) [6]. There is strong evidence of the reduction of post-operative wound complication rates in orthopaedic, vascular surgery, and abdominal surgery with the implementation of CINPT [7]. However, research on CINPT in breast surgery is still in its infancy, with few institutions implementing it as part of post-operative wound management.

To assess the benefits of CINPT in breast surgery, we conducted a systematic review looking into post-operative wound complications in breast surgery in patients treated with CINPT as compared to SOC. Furthermore, we investigated the overall cost-savings and improved quality of life with CINPT as compared to SOC.

This publication will be presented at the Royal Australasian College of Surgeons, Annual Scientific Conference 2022 to be held in Brisbane, Australia, as a verbal presentation on May 4, 2022.

## Review

Methods

Search Strategy

A comprehensive search of PubMed, Medline, and Google Scholar for English language studies was conducted using the following keywords: (1) Negative Pressure Wound Therapy, (2) Closed Incision Negative Pressure Therapy, (3) Breast Surgery, (4) Surgical Site Infections, and (5) Wound Complication. The reference lists were reviewed for additional pertinent studies. Studies that referenced negative pressure wound therapy were taken as CINPT if the study stated that the negative pressure dressing was used over a closed incision.

Study Selection

This systematic review takes guidance from the Preferred Reporting Items for Systematic Review and Meta-analysis Protocols (PRISMA-P) [8]. Published English language reports that encompassed our keywords were included. We reviewed several outcomes including (1) evaluating the risk of surgical site infections in patients undergoing breast surgery with the utilization of CINPT compared to a control group that used SOC as post-operative wound management, (2) cosmetic outcomes of patients who had CINPT, and (3) cost savings associated with CINPT.

Data extracted from the studies included the publication year, country, study characteristics, individual study database, and type of CINPT or SOC used. The data were extracted by two authors (AL, JK).

Study Outcomes

Our primary objective was to determine the risk of post-operative complications in breast surgery patients receiving CINPT as compared to SOC. Other outcomes included the differences in cost savings acquired by the hospital and the cosmetic outcomes between CINPT and SOC.

Results

Literature Review

**Figure 1:**
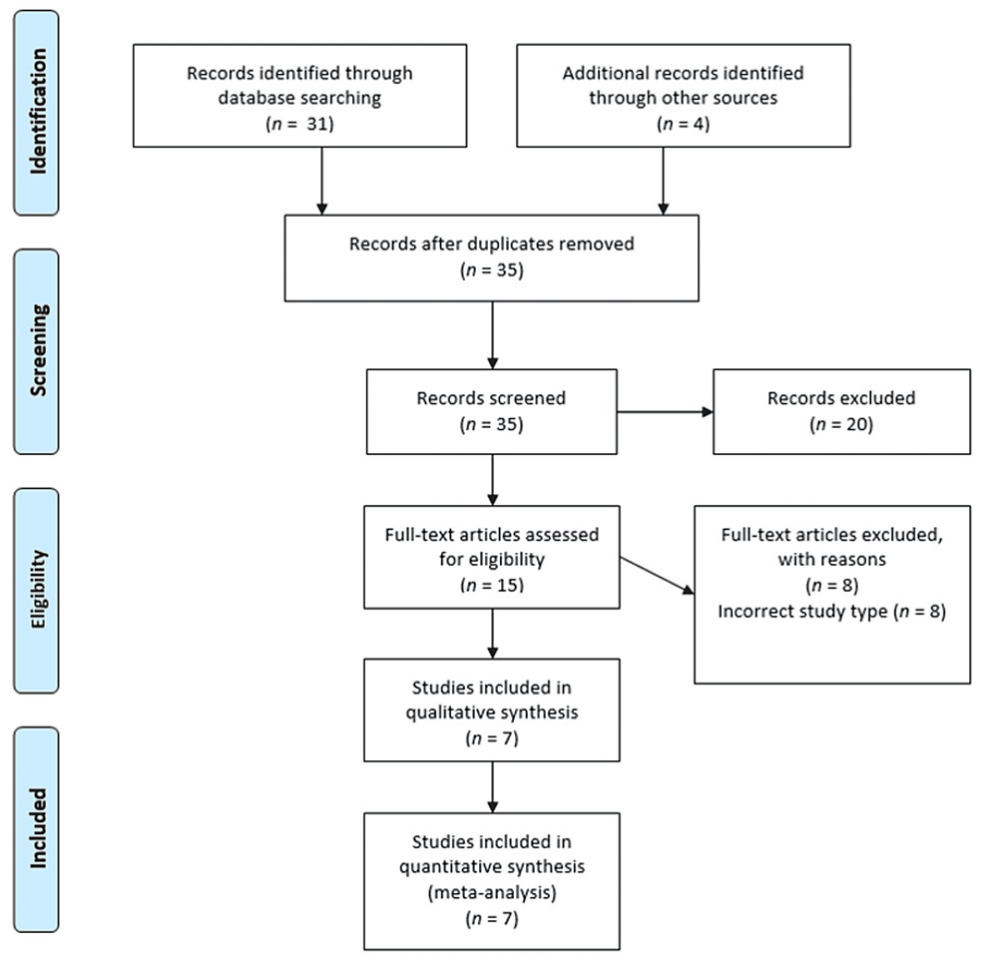
Study selection for CINPT vs SOC according to PRISMA-P CINPT: closed incision negative pressure therapy; SOC: standard of care dressings; PRISMA-P: Preferred Reporting Items for Systematic Review and Meta-analysis Protocols

From the initial analysis, 35 studies were identified with an additional four studies added after reviewing the references of the initial studies. Fifteen studies were assessed for eligibility but only seven were found eligible after qualitative synthesis (Figure 1). A total of 691 patients were included in the CINPT group as compared to 829 patients in the SOC group. The breast surgeries included: (1) mastectomies (total, skin sparing, nipple sparing, (2) with or without sentinel node biopsies, (3) with or without axillary clearance, (4) with or without immediate breast reconstruction, and (5) breast reduction mammoplasties. The characteristics of the seven studies are shown in Table 1.

**Table 1:** Characteristics of selected studies CINPT: closed incision negative pressure therapy; SSE: surgical site infections; SOC: standard of care dressings; BCS: breast-conserving surgery PICO: single-use negative pressure wound therapy device (Smith & Nephew Medical Limited, Kingston upon Hull, United Kingdom); PREVENA™: disposable powered negative pressure system designed specifically for the management of closed surgical incisions (3M Company, Saint Paul, Minnesota, United States)

Study (Author, Year, Country)	Background	N (CINPT)	N (SOC)	Intervention	Complications	Procedure	Follow-Up
Pellino, 2014, Italy [9]	Efficacy of PICO in preventing SSE compared with conventional dressings in breast surgery patients	25	25	CINPT: PICO	Seroma, infectious SSE according to CDC criteria, Global Asepsis Score	-	3, 7, 30 days and at 3 months
Kim, 2016, South Korea [10]	Use of CINPT vs SOC	45	183	-	Mastectomy flap necrosis, Infection, Seroma, Haematoma, Expander explantation	Post mastectomy immediate expander based reconstruction	30 days post-operative follow-up
Galiano, 2018, USA [11]	Efficacy of PICO compared to SOC	200	200	CINPT: PICO	Infection, Dehiscence or delayed healing (not 100% closed within 7 days of the first surgical procedure)	Bilateral reduction mammoplasty	21 days post-operative follow-up
Gabriel, 2018, USA [12]	Efficacy of PREVENA vs SOC in immediate breast reconstruction	331	334	CINPT: PREVENA™	Wound complication, infection, haematoma/seroma, breast pain, fat necrosis, radiation, pneumonitis, rib fracture graft/implant complication, and implant explantation	Mastectomy with immediate expander-based breast reconstruction	3 months post-op follow-up
Ferrando, 2018, Italy [13]	Efficacy of PREVENA vs SOC dressing for oncological breast surgery	25	22	CINPT: PREVENA™	Infection, seroma/haematoma, skin necrosis	BCS, total mastectomy, skin-sparing mastectomy, nipple-sparing mastectomy, autologous flap	7, 14, 30, 90 days and 12 months follow-up
Tanaydin, 2018, Netherlands [14]	Number of wound healing complications within 21 days when comparing CINPT with fixation strips	32	32	CINPT: PICO	Superficial wound dehiscence, scar quality, and aesthetic appearance	Bilateral breast reduction mammoplasty	21, 42, 90, 180, and 365 days
Larsen, 2020, Denmark [15]	Effect of CINPT on seroma formation who underwent mastectomy	33	33	CINPT: PICO	Seroma, infection, skin necrosis	Mastectomy and sentinel lymph node biopsy; mastectomy and axillary clearance	3, 5, 7, 10, 13, 16, 19 days

Devices

From our systematic review, two CINPT devices were found to be mainly used in post-operative wound management for breast surgery. This included (1) PICO (Smith & Nephew Medical Limited, Kingston upon Hull, United Kingdom). This is a portable single-use (disposable after seven days) CINPT device that delivers -80mmHg of negative pressure over a closed incision and (2) PREVENA™ (3M Company, Saint Paul, Minnesota, United States). This is a single-use therapy unit, applied for seven days that delivers -125mmHg of negative pressure over a closed incision and has a replaceable 45ml exudate canister. The study by Kim et al. did not specify what CINPT was used. It only stated a negative pressure of -125mmHg was applied over the closed incision with a polyurethane sponge for three days.

Post-operative SOC mainly comprised Steri-Strips and a variety of simple waterproof dressings such as DuoDERM® (ConvaTec Group plc, Deeside, United Kingdom) or OPSITE.

Surgical Site Infection

The overall rates of SSI showed a general trend in favour of CINPT as compared to SOC, with post-operative SSI rates of 1-21% (Table 2). Most studies reported a post-operative rate of 1-8%. A significant outlier was reported in Larsen et al., where 21.4% of patients developed an SSI after mastectomy and axillary clearance (three out of 14 patients) (Table 2).

**Table 2:** Comparison of surgical site infections in CINPT and SOC *Exact number not specifically stated CINPT: closed incision negative pressure therapy; SOC: standard of care dressing

Study (Author, Year, Country)	CINPT	SOC	P-value
Pellino, 2014, Italy [9]	2/25 (8%)	9/25 (36%)	0.04
Kim, 2016, South Korea [10]	1/45 (2.2%)	5/183 (2.7%)	1.00
Galiano, 2018, USA [11]	4/200 (2.0%)	6/200 (3.0%)	0.532
Gabriel, 2018, USA [12]	7/331 (2.1%)	15/334 (4.5%)	0.0225
Ferrando, 2018, Italy [13]	1/25 (4%)	7/22 (31.8%)	N/A
Tanaydin,2018, Netherlands [14]	*	*	0.001
Larsen, 2020, Denmark [15]	Mastectomy and sentinel node biopsy: 0/19 (0%); mastectomy and axillary clearance: 3/14 (21.4%)	Mastectomy and sentinel node biopsy: 1/19 (5.3%); mastectomy and axillary clearance: 0/14 (0%)	N/A

Cosmetic Outcomes

Our literature review revealed that cosmetic outcome from CINPT is superior to SOC. This was based on The Patient Scale and Observer Scale (POSAS) and Manchester Scar Assessment Scale. Both these scales are questionnaires filled out by both patients and practitioners based on the cosmetic outcomes from surgery. Tanaydin et al. [14] included 32 patients in each arm while Ferrando et al. [13] included 25 patients in the CINPT arm and 22 in the SOC arm. Both studies reported a clinically significant difference in both observer and patient-based cosmetic outcomes, with better scarring in the CINPT group one-year post surgery.

Summary Review of Risk of Bias

All seven studies underwent a Cochrane review of risks of bias by two authors (AL, JK). The results of the risks of bias are shown in Table 3. In general, because of the nature of the study, there was no blinding of participants or personnel. Furthermore, there was no blinding of the outcome assessments as these patients were reviewed by the same physicians in the post-operative clinics. However, attrition bias was kept to a minimum in all studies as the follow-up periods were short.

**Table 3:** Cochrane review of risks of bias

Study (Author, Year)	Random sequence generation (selection bias)	Allocation concealment (selection bias)	Blinding of participants and personnel (performance bias)	Blinding of outcome assessment (detection bias)	Incomplete outcome data (attrition bias)
Pellino, 2014 [9]	+	N/A	N/A	N/A	+
Kim, 2016 [10]	?	N/A	N/A	N/A	+
Galiano, 2018 [11]	+	N/A	N/A	N/A	+
Ferrando, 2018 [13]	-	N/A	N/A	N/A	+
Gabriel, 2018 [12]	?	N/A	N/A	N/A	+
Tanaydin, 2018 [14]	+	N/A	N/A	N/A	+
Larsen, 2020 [15]	+	N/A	N/A	N/A	+

Discussion

Wound care is a vital component in breast surgery, particularly the reduction of adverse wound outcomes. Preventing SSI is important as it may delay time to adjuvant therapy or reconstruction management and affect patients’ quality of life and satisfaction. There are several risk factors involved in the development of SSI in breast surgery including co-morbidities such as obesity and diabetes mellitus, along with iatrogenic factors such as operative sterility and post-operative chemoradiotherapy in oncological patients.

CINPT has been available for the last two decades as an alternative method of managing complex surgical wounds, especially in patients who are at high risk of developing wound dehiscence [5]. The negative pressure from vacuum-assisted closure of wound therapy that is applied to close incision is aimed at promoting blood flow to the wound edges, removing infectious debris, reducing tissue oedema, and reducing the tension of the suture line [16]. The devices that were used in breast surgery, PREVENA and PICO, have been widely adopted by other surgical subspecialties including orthopaedic, gynaecological, colorectal, and vascular [17]. The application of CINPT has the potential to reduce post-operative SSI by 63% and reduce the length of hospital stay [18]. Despite this evidence, its use in breast surgery remains low and far between, with very limited evidence regarding its use.

We believe that this is the first systematic review that examines the use of CINPT in breast surgery, including mastectomies and axillary dissections. Our review has shown that CINPT can have a positive effect in terms of reducing wound complication rates, better cosmetic outcomes, and better cost-effectiveness as compared to SOC. The use of CINPT has been shown to decrease wound dehiscence and SSI rates by 50% in abdominal wall surgery patients with co-morbidities including obesity (BMI > 30kg/m2), diabetes mellitus, and smoking [19,20]. It can be extrapolated that post-operative complications in patients with these co-morbidities would be reduced in breast surgeries with long incisions (e.g mastectomies or wise-pattern mammoplasty) given the similarities in the operative fields. The use of CINPT is particularly useful in large wounds with its ability to hold incision sites together, remove excess fluids, enhance tissue growth, and the prevention of external contamination [21]. This is especially applicable to major breast surgeries including mastectomies and large volume mammoplasties. Animal studies conducted had shown that CINPT used on operative fields with these characteristics had the strongest tensile strength at seven and 21 days as compared to a control group (24.6N at seven days and 61.67N at 21 days). Furthermore, CINPT allows for increased blood perfusion into the incision site as compared to the SOC group, encouraging greater healing capacity [6].

This is evident in the studies where a simple mastectomy and sentinel node biopsies were performed for oncological reasons [13,15]. This result was similarly replicated in patients who had reduction mammoplasties [14]. It is interesting to note that post-operative wound infection rates reported by Larsen et al. were significantly higher in patients who were in the CINPT group after undergoing a mastectomy and axillary clearance, and this was the only study that investigated the application of CINPT post axillary clearance. It is theorised that an axillary clearance can lead to the formation of a large volume seroma secondary to the disrupted lymphatics, and this increases the risk of post-operative wound infections [15]. However, why it was higher than the SOC group in the study has yet to be explained.

Our review has also reported the benefits of CINPT in immediate breast-reconstruction patients. With an increasing number of patients opting to have breast reconstruction after oncological breast surgery, cosmetic outcomes including scarring are a major factor in the patient’s overall quality of life. Several studies had reported a clinically significant decrease in overall wound complication rates including skin necrosis, surgical site infections, and seroma [10,12,13]. A reduction in post-operative wound complication rates will improve the post-mastectomy cosmetic outcome for the patient. Currently, Kim et al. had stated the benefits of CINPT in prosthetic breast reconstruction [10]. While Ferrando et al. included patients with both autologous flap reconstructions and implant reconstructions, the study did not stratify wound complications between the type of reconstruction [13]. However, the type of breast reconstruction was not clearly stated by Gabriel et al. [12]. Because of the small sample size collected for wound complications in immediate reconstruction, further studies are required to determine the specific sort of breast reconstruction surgery that will benefit from CINPT.

CINPT has also the potential benefit of reducing hospital costs by reducing the post-operative wound complication rates in patients. SSI is a common post-surgical complication and can result in a substantial economic burden - contributing to prolonged hospital admissions and USD1,600,000 in additional cost per year [22]. Studies have shown that the use of CINPT has the potential for reducing health care costs postoperatively [12]. Despite CINPT being considerably more expensive compared to SOC, it is more cost-effective due to decreased risk of infections and complications [12,15]. Readmission and reoperation rates have been assessed to be lower in patients treated with CINPT compared to control groups following abdominal wall constructions [23]. Economic analysis has demonstrated cost savings of USD218 per patient with the use of CINPT compared to SOC after breast reconstructions, including costs of therapy and complications [12]. An international consensus conference in 2016 provided further evidence that CINPT appears to have the potential to reduce surgical costs of up to USD9,000 per patient in high-risk patients in a broad range of high-risk surgical procedures [17]. However, a Cochrane review in 2020 produced varied results on the cost-effectiveness of CINPT amongst a wide range of surgeries [24]. Hence, further evaluations of cost-effectiveness in diverse patient risk populations and large cohort studies of CINPT in post-operative breast surgery are therefore warranted.

We acknowledge that there are several limitations to our systematic review and the technical use of the system itself. First, we acknowledge the small number of studies included in our systematic review. As CINPT in breast surgery is still in its infancy, there are few studies available that evaluate this topic. Furthermore, the sample size for some of these studies was small. We also recognise that there was a degree of intra-study variability and heterogeneity. Although these studies specifically investigated breast surgeries, the specific characteristics of these studies were variable. For example, some studies assessed skin-sparing mastectomies while others investigated total mastectomies. Furthermore, post-operative follow-up rates varied between these selected studies. Patients should be followed up in about six to six months post-surgery to assess their response to their cosmetic outcome. It should also be noted that three of the seven studies had some form of interest in the companies that supplied their CINPT and this can pose a risk of bias for CINPT [11,12,15].

The application of CINPT itself can be quite laborious. As it works on a sealed system, any leakage will cause the CINPT to lose suction. Hence, patients need to maintain caution to keep the system dry and avoid inadvertently peeling the dressing of the incision site. Furthermore, based on our experience, simple mastectomies can produce up to 500mls of serous exudate post surgery. Some of the drain tube canisters that are attached to the CINPT dressings can only hold up to 50mls and patients are required to constantly change the canisters if a large volume of seroma is absorbed.

## Conclusions

CINPT has the potential to reduce post-operative wound complications in breast surgery and have better cost effectiveness as compared to SOC. Furthermore, there is the potential for better cosmetic outcomes as compared to SOC as reported in our systematic review. This can translate to an overall better quality of life for patients, especially those undergoing oncological breast surgery. However, we acknowledge CINPT in breast surgery is still in its infancy, and these are early trends. Hence, further large prospective studies are recommended to define the efficacy of CINPT in breast surgery.
